# GSK3-beta as a candidate therapeutic target in soft tissue sarcomas

**DOI:** 10.1186/s13045-021-01215-x

**Published:** 2021-12-02

**Authors:** S. Verbeke, R. Perret, V. Chaire, E. Richard, V. Velasco, F. Giles, L. Cavalcante, A. Italiano

**Affiliations:** 1 Sarcoma Unit, Institut Bergonié, 229 cours de l’Argonne, 33000 Bordeaux, France; 2 INSERM, U1218, Bordeaux, France; 3 Department of Pathology, Institut Bergonié, Bordeaux, France; 4 Actuate Therapeutics, Fort Worth, TX USA; 5 Faculty of Medicine, University of Bordeaux, Bordeaux, France

**Keywords:** Glycogen synthase kinase 3β, Soft tissue sarcomas, 9-ING-41

## Abstract

Soft tissue sarcoma (STS) is a predominantly fatal rare malignancy with inadequate treatment options. Glycogen synthase kinase 3β (GSK-3β) is an emerging target in human malignancies. Its therapeutic relevance in STS is unknown. We analyzed the prognostic impact of GSK-3β gene and protein expression in two independent cohorts of patients with STS. We then treated STS cell lines and mice xenografts with a novel GSK-3 inhibitor 9-ING-41 alone or in combination with chemotherapy. We demonstrated that 9-ING-41 treatment induced significant STS cells apoptosis and was synergistic in vivo when combined with chemotherapy. Mechanistically, 9-ING-41 induces significant apoptosis of STS cells via suppression of NF-κB-mediated X-linked inhibitor of apoptosis protein (XIAP) expression. These data support the inclusion of patients with STS in clinical studies of 9-ING-41 alone and in combination with chemotherapy.

**Supplementary Information:**

The online version contains supplementary material available at 10.1186/s13045-021-01215-x.

To the Editor

The prognosis of patients with advanced STS is extremely poor with a median overall survival of less than 18 months [1, 2]. Identification of new therapeutic strategies is therefore an important medical need.

Glycogen synthase kinase-3β (GSK-3β) has been shown to play an important role in tumor progression particularly through the modulation of oncogenes, cell cycle regulators and mediators of epithelial–mesenchymal transition [3]. Recent studies have also demonstrated that aberrant overexpression of GSK-3β promotes tumor growth and chemotherapy resistance in various solid tumors including pancreatic, colorectal and prostate cancer through differential effects on pro-survival NF-κB and c-Myc pathways as well as on TNF-related apoptosis-inducing ligand (TRAIL) and p53-mediated apoptotic mechanisms [4–6]. GSK-3β represents therefore an important therapeutic target in human malignancies.

9-ING-41 is a small molecule potent selective GSK-3β inhibitor with antitumor activity in several epithelial tumor models as shown by the interim results of the first clinical trial reported in patients with refractory cancers. Five other phase 2 clinical trials are ongoing in salivary gland carcinoma, myelofibrosis, pancreatic adenocarcinoma and pediatric patients with advanced malignancies (https://clinicaltrials.gov/ct2/results?cond=&term=9ING41&cntry=&state=&city=&dist =) [7–9]. We report here the first study investigating the therapeutic potential of GSK-3β targeting with 9-ING-41 in STS (see Additional file 1: Methods).

We first used the Gene Expression Profiling Interactive Analysis (GEPIA), a web-based interactive database that compiles the standardized analysis of RNA-Seq data from 9736 tumors and 8587 normal samples based on The Cancer Genome Atlas (TCGA) and Genotype-Tissue Expression (GTEx) databases. We found that high *GSK3β* gene expression was significantly associated with worse disease-free survival (log-rank test, p = 0.0088, Additional file 1: Fig. 1). To confirm the prognostic value of GSK3β expression, we analyzed by immunohistochemistry a series of 402 patients with STS (Fig. 1A and Additional file 1: Tables 1 and 2). We observed positive expression of GSK3β in 220 (54.7%) cases. High expression of GSK3β (> 50% of tumor cells) was significantly associated with poor prognosis (median metastases-free survival 139 months versus not reached, p = 0.02, Fig. 1B) supporting the hypothesis that GSK3β may represent a potential therapeutic target. As shown in Fig. 1C, GSK3β protein expression provided extra information to further refine the prognosis of patients besides histological grade which is considered the most significant predictor of outcome.

**Fig. 1:**
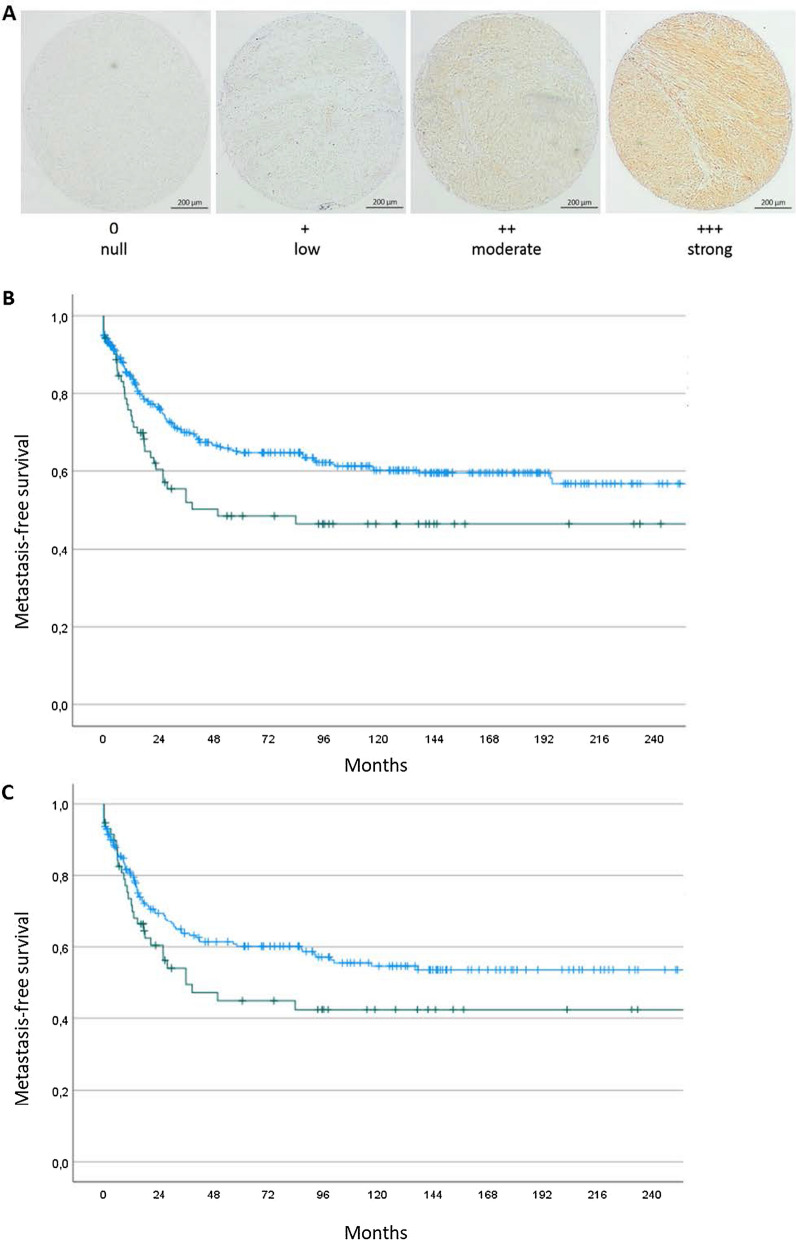
**A** Examples of GSK3β staining intensity in soft tissue sarcomas. GSK3β was evaluated semi-quantitatively including percentage (0–100%) and intensity (0 = null, 1 = low, 2 = moderate, 3 = strong). **B** Kaplan–Meier curves of metastases-free survival in 402 patients with soft tissue sarcomas according to GSK3β expression (blue line: low expression, < 50% of tumor cells *n* = 296; green line: high expression: ≥ 50% of tumor cells *n* = 106). **C** Kaplan–Meier curves of metastases-free survival in 268 patients with grade 3 soft tissue sarcomas according to GSK3β expression (blue line: low expression, < 50% of tumor cells *n* = 200; green line: high expression: ≥ 50% of tumor cells *n* = 68)

9-ING-41 is a first-in-class, maleimide-based small molecule potent selective GSK3β inhibitor that has recently entered into clinical development. To examine its antitumor effect on STS, a panel of 20 STS cell lines were plated and treated with increasing concentrations of 9-ING-41 for 72 h. We showed that pharmacological inhibition of GSK3β by 9-ING-41 suppressed the viability of all the 20 STS cell lines encompassing several histological subtypes with IC50 values ranging from 0.1 to 0.6 µM (Additional file 1: Table 3, Additional file 1: Fig. 2A).

Then, we demonstrated that 9-ING-41 suppresses cell viability through induction of apoptosis in a large panel of STS in vitro by using annexin V/PI, and immunoblots of apoptotic markers such as phospho-γH2AX, cleaved-Caspase-3 and cleaved-PARP confirming the phenotype of significant cell death in 9-ING-41-treated cells (Additional file 1: Figs. 2B and 3).

GSK3β controls many cellular process including the survival NF-kappaB pathway. Therefore, we assessed by Western blot the activation of the IκB kinase (IKK), a key protein involved in the translocation of NF-kappaB to the nucleus. As shown in Additional file 1: Fig. 4, the phosphorylation of IKKα/β at serine 176/177 is decreased after 24 h of 9-ING-41 treatment in different STS cell lines suggesting an inhibition of the NF-κB pathway. To confirm these results, the expression of three NF-κB target genes involved in anti-apoptotic effect, *Bcl-2, Bcl-XL and XIAP*, was examined. Although immunoblots revealed no significant changes in expression of Bcl-2 and Bcl-XL, XIAP expression was reduced in almost all cell lines tested (Additional file 1: Fig. 4). These data suggest that 9-ING-41 induces apoptosis in STS cell lines through the inhibition of NF-κB pathway and a subsequent decreased expression of the anti-apoptotic gene *XIAP*.

We next evaluated the effect of 9-ING-41 in vivo by using the IB115 liposarcoma model. Mice were randomized in 4 groups and treated by intraperitoneal injection with vehicle, 9-ING-41, doxorubicin (the standard of care for STS) or combination of both drugs. As shown in Fig. 2, the tumors expanded aggressively in vehicle-treated animals, whereas in monotherapy a slight decrease of tumor growth was observed for 9-ING-41- or doxorubicin-treated mice. On the other hand, the combination treatment caused a significant decrease in tumor growth suggesting that 9-ING-41 can potentiate the effect of doxorubicin even with a low dose of chemotherapy without significant signs of toxicity as shown by the stable body weight of the mice (Fig. 2B).

**Fig. 2:**
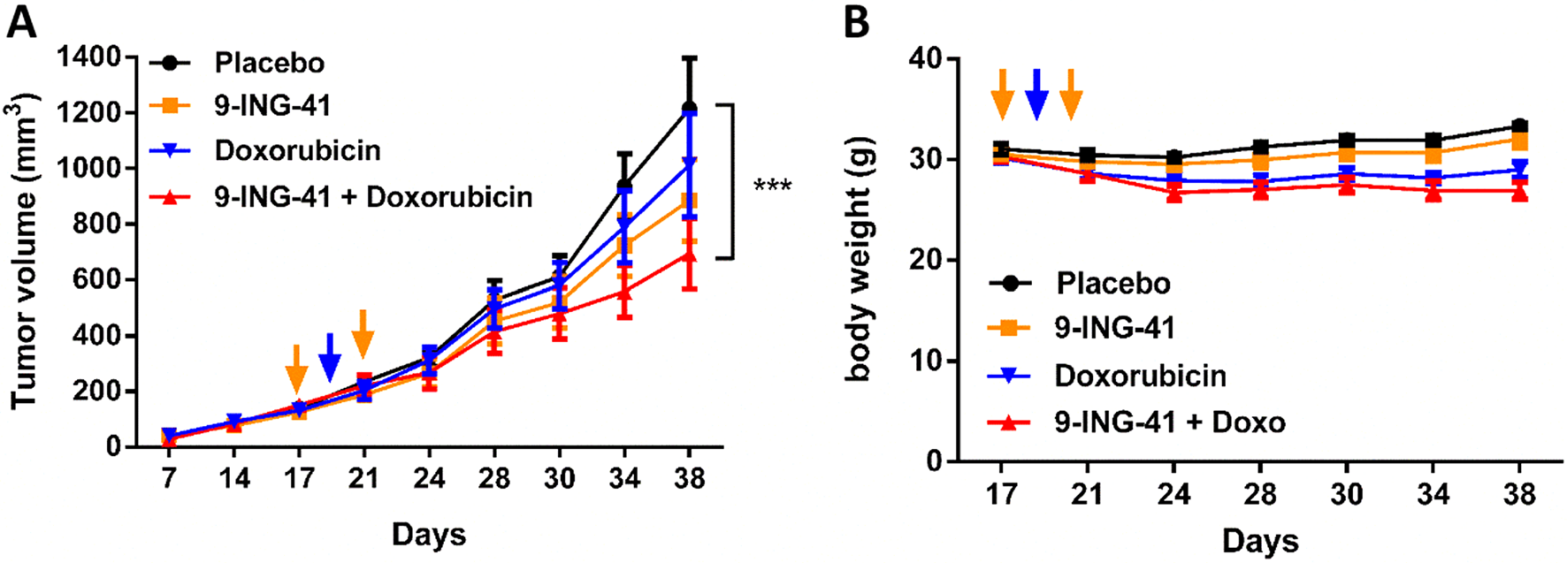
In vivo effect of GSK3β inhibitor 9-ING-41 in soft tissue sarcoma. IB115 liposarcoma cells were xenografted in NSG mice. Once tumors reached 100mm^3^ (day 17), mice were treated either with placebo (9 mice) or with two injections of 9-ING-41 at 70 mg/kg (orange arrow, 7 mice) or one injection of Doxorubicin at 1 mg/kg (blue arrow, 6 mice) or both drugs (6 mice). Tumor growth (**A**) and mice body weight (**B**) were monitored until day 38 and analyzed with GraphPad prism software using two-way ANOVA test and Bonferroni post-hoc test (***p < 0.001). Combination treatment of 9-ING-41 and doxorubicin reduces significantly tumors volume

By analyzing two large independent data sets, we show here than GSK3β expression both at the gene and protein expression level is associated with increased risk of metastatic relapse and adverse outcome in STS patients suggesting an important role in sarcoma tumorigenesis and a potential role as a therapeutic target. Moreover, instead of using the commercially available toolkit GSK-3β inhibitors, which are not amenable for clinical studies, we decided here to investigate 9-ING-41, a first-in-class GSK-3β inhibitor which has recently entered clinical development in cancer patients. Preclinical studies have shown that 9-ING-41 induces significant apoptosis of cancer cell survival via suppression of NF-κB-mediated B cell lymphoma 2 (Bcl-2) and XIAP expression in leukemia and solid tumors [10, 11]. The NFκB transcription factor family is a highly conserved group of proteins playing an important role in the regulation of the cell physiology such as differentiation, apoptosis and survival. Pharmacological NFκB inhibition has been shown to reduce cell growth in a spectrum of soft tissue sarcomas [12]. Here, we have shown that 9-ING-41 led to a decreased expression of the antiapoptotic molecule, XIAP, and resulted in an increased apoptosis as shown by PARP cleavage and caspase activation assay in STS cells. This suggests that 9-ING-41 may represent a candidate for the targeted therapy of STS. 9-ING-41 is currently under investigation as a single agent and in combination with chemotherapy in cancer patients with advanced disease [13]. Preliminary results have shown a good safety profile and promising early signs of clinical activity in several solid tumors including melanomas and pancreatic cancers. Based on these results, a clinical trial investigating 9-ING-41 in sarcoma patients will be conducted.

### Supplementary Information

**Additional file 1:** Supplementary methods and results.

## Data Availability

Source data are available on request to the corresponding author.

## Abbreviations

GEPIA: Gene Expression Profiling Interactive Analysis
GSK-3β: Glycogen synthase kinase 3β
GTEx: Genotype-Tissue Expression
IKK: The IκB kinase
TCGA: The Cancer Genome Atlas
TRAIL: TNF-related apoptosis-inducing ligand
STS: Soft tissue sarcomas
XIAP: X-linked inhibitor of apoptosis protein
