# Supplementary material for: GSK3-beta as a candidate therapeutic target in soft tissue sarcomas
Source: J Hematol Oncol. 2021 Dec 2;14:202. doi: 10.1186/s13045-021-01215-x (PMC8641200; doi:10.1186/s13045-021-01215-x)
Supplement: Supplementary file 1 — Additional file 1: Supplementary methods and results. [file 13045_2021_1215_MOESM1_ESM.docx]

**SUPPLEMENTARY METHODS AND RESULTS**

SUPPLEMENTARY METHODS

Cell culture and Reagents

The soft-tissues sarcoma (STS) cell lines used in this study called “IBxxx” or JR588 and KN473 were derived from human STS surgical specimens after obtaining written, informed patient consent and Bergonié Institute Institutional Review Board approval. Each home-made cell line was characterized by array comparative genomic hybridization every 10 passages until p50 to verify that its genomic profile was still representative of the originating tumor sample. No drift in the cell line maintenance or genetic imbalances were shown along passages. 93T449 (RRID: CVCL_U614) and 94T778 (RRID:CVCL_U613) liposarcoma cell lines were kindly provided by Dr Laurence Bianchini and MON cell line (RRID:CVCL_M846) by Dr Francois Le Loarer. VAESBJ (ATCC Cat# CRL-2138, RRID:CVCL_1785), G401 (ATCC Cat# CRL-1441, RRID:CVCL_0270), G402 (ATCC Cat# CRL-1440, RRID:CVCL_1221), A204 (ATCC Cat# CRL-7900, RRID:CVCL_1058) STS cell lines were purchased from ATCC . Cells were grown in RPMI medium 1640 GlutaMAX^TM^ Supplement (Life Technologies, Carlsbad USA) in the presence of 10% (v/v) fetal bovine serum and Penicillin/Streptomycin 1% (Thermo Scientific, Gibco^TM^), in flasks. Cells were maintained at 37°C in a humidified atmosphere containing 5% CO2. Cells were routinely passaged every 3 days and all the experiments were performed with cell lines between passages 25 and 60.

9-ING-41, a potent glycogen synthase kinase-3 beta (GSK-3β) inhibitor was provided by Actuate Therapeutics Inc. (Fort Worth, TX, USA) and prepared as a 10 mM stock solution in DMSO and stored at -20°C for in vitro studies.

Tissue Micro Array and immunohistochemistry

Tissue Micro Arrays (TMA) were produced from Formalin-Fixed Paraffin-Embedded (FFPE) tumor blocks of 403 patients with surgically resected MFH using a Tissue Arrayer MiniCore 3 (Excilone). Three representative spots of 1-mm cores of tumor sample were assessed. Immunohistochemistry was performed on four micrometers paraffin sections as per standard technique on a Ventana Benchmark ULTRA automat (Roche, Bâle, Switzerland) using Monoclonal primary antibody for GSK3β (Cell Signaling Technology Cat# 12456, RRID:AB_2636978). Amplification and detection steps were performed with an Ultraview kit and 3,3'-diaminobenzidine was used as a chromogen. GSK3β staining on TMA slides was evaluated semi-quantitatively by a trained pathologist (Raul Perret) blinded of clinical data. Results were scored by multiplying the percentage of positive cells (P) by the intensity (0 = null, 1 = low, 2 = moderate, 3 = strong) of staining on tumor spots

Cell viability assay

Depending on the doubling time, cells were seeded in triplicate at 250 to 2000 cells/well into 384-well plates, cultured with fresh growth medium for at least 24 hours and treated with a range of increasing concentrations of 9-ING-41 (1nM to 24µM) for 72 hours. Cell viability was assessed by staining viable cells with the cell-permeant SYTO 24 green fluorescent probe (Thermo Fisher Scientific #S7559) and apoptotic cells with propidium iodide during 30 minutes at 37°C. Fluorescent viable and apoptotic cells were measured by Cytation™ 3 Cell Imaging Multi-Mode Reader and analyzed by Gen5 software (RRID:SCR_017317). The half maximal inhibitory concentration (IC50) was calculated with GraphPad Prism software version 5.0 for Windows (GraphPad Software, RRID:SCR_002798, La Jolla, USA). Each experiment was repeated at least 3 times.

Determination of Apoptosis

STS cells (4000 cells/well) were seeded in 96-well plates in triplicate. After 24h, cells were treated for 48 hours with 0.5 µM of 9-ING-41. After treatment, cells were washed once with phosphate-buffered saline (PBS) and labeled with annexin-V-FITC and propidium iodide (PI) according to the manufacturer’s protocol (BD Biosciences, San Jose, CA, USA). Then, apoptosis was determined by fluorescence-activated cell-sorting (FACS Calibur flow cytometer, BD Biosciences, San Jose, CA, USA) analysis of annexin-V-FITC and PI labelling. The percentage of cells in early apoptosis (annexin-V-positive, PI-negative) and in late apoptosis or necrosis (annexin-V and PI-positive) was calculated using FlowJo version 7.6.3 for Windows (Tree Star Inc, Ashland, OR, USA). The percentages of overall death (sum of early and late apoptosis) are represented as the mean ± SEM values based on 3 independent experiments.

Western blot

Cells were treated with or without 5µM of 9-ING-41 for 24h. Floating and attached cells were harvested in 100 μl of radio-immuno-precipitation assay (RIPA) lysis buffer. The lysate was centrifuged (13 000 rpm, 15 min, 4°C), and the supernatant was stored at -20°C. Equal amounts of total protein (30 μg) were electrophoresed on 12% or 8% sodium dodecyl sulfate polyacrylamide gels and transferred onto polyvinylidene difluoride membranes. The blots were probed overnight at 4°C with an anti- XIAP (BD Biosciences Cat# 610716, RRID:AB_398039), anti-actin (Sigma-Aldrich Cat# A3853, RRID:AB_262137), anti-phospho-γH2AX (Cell Signaling Technology Cat# 9718, RRID:AB_2118009), anti- γH2AX (Cell Signaling Technology Cat# 7631, RRID:AB_10860771), Phospho-IKK-alpha (Ser176)/IKK-beta (Ser177) (Cell Signaling Technology Cat# 2078, RRID:AB_2079379), IKK-beta (Cell Signaling Technology Cat# 2678, RRID:AB_2122301), Bcl-XL (Cell Signaling Technology Cat# 2764, RRID:AB_2228008), Bcl2 (Cell Signaling Technology Cat# 15071, RRID:AB_2744528), cleaved Caspase-3 (Cell Signaling Technology Cat# 9664, RRID:AB_2070042), PARP (Cell Signaling Technology Cat# 9542, RRID:AB_2160739) primary antibody diluted in PBST (DPBS 10X (Gibco^TM^) after 1X dilution; 0.1% Tween-20) with 5% bovine serum albumin. The horseradish peroxidase-conjugated secondary antibody (Santa Cruz Biotechnology Inc. Dallas, USA) was diluted 1:5000. Bound antibodies were visualized on Fusion Fx7 imaging system (Fisher Bioblock Scientific, Waltham, USA) using the Immobilon^TM^ Western enhanced chemiluminescence detection kit (Millipore Corporation, Billerica, USA). The resulting bands were analyzed and quantified using ImageJ 1.48v software (RRID:SCR_003070, National Institutes of Health, Bethesda, USA).

Animal study

All animal experiments were performed with the approval of the institutional animal use and care committee under project license APAFiS #17900-2018112722234037. This study followed the French and European Union guidelines for animal experimentation (RD 1201/05, RD 53/2013 and 86/609/CEE, respectively). IB115 cells (3 × 10^6^ cells/200 µL) were inoculated subcutaneously into the right flank of 6- to 8-week-old NSG male mice which were procured and housed in the institutional animal facilities. Once palpable, tumor volumes were calculated using the following formula: length × width^2^/2. Two weeks after cells implantation when the average size of the tumors was around 100 mm^3^, mice were randomized into 4 groups and treated by intraperitoneal injection with: Vehicle (DMSO, 9 mice), Doxorubicin (1 mg/kg, 6 mice, one injection), 9-ING-41 (70 mg/kg, 7 mice, two injections), or both Doxorubicin and 9-ING-41 (6 mice). Tumor size was measured every 2-3 days with calipers until the end of the study (day 38), then mice were euthanized. Tumor progression was analyzed with GraphPad Prism software using two-way ANOVA test and Bonferoni post-hoc test.

Statistics

For analysis of metastases-free survival according to expression levels of GSK3β, the cut-off date for statistical analysis of baseline demographic data and clinical outcome was the 03/31/2018. Survival rates were estimated using the Kaplan–Meier method. Analyses were performed using SPSS 18.0 statistical software (IPSS Inc., Chicago, USA). All statistical tests were two-sided, and p < 0.05 indicated statistical significance.

SUPPLEMENTARY RESULTS

**SUPPLEMENTARY TABLE 1 :** Characteristics of the study population (n=402)

| **Characteristics** | | **Patients** |
| --- | --- | --- |
| **Sex** |  |  |
|  | Female | 194 (48.3%) |
|  | Male | 208 (51.7%) |
| **Age** |  |  |
|  | median (range) | 63 (19 - 95) |
| **Location of tumor** | |  |
|  | Upper/Lower limb | 281 (69.9%) |
|  | Trunk wall | 73 (18.2%) |
|  | Internal trunk | 41 (10.2%) |
|  | Others | 7 (1.7%) |
| **Depth of tumor** | |  |
|  | Deep | 371 (89.4%) |
|  | Superficial | 44 (10.6%) |
| **Size of tumor** | |  |
|  | ≤ 5 cm | 95 (23.8%) |
|  | > 5 cm and ≤ 10 cm | 159 (39.8%) |
|  | > 10 cm | 148 (36.3%) |
| **Histological type** | |  |
|  | Undifferentiated pleomorphic sarcoma | 124 (30.8%) |
|  | Leiomyosarcoma | 143 (35.6%) |
|  | Myxofibrosarcoma | 64 (15.9%) |
|  | Liposarcoma - dedifferentiated | 21 (5.2%) |
|  | Pleomorphic rhabdomyosarcoma | 25 (6.2%) |
|  | Others | 25 (6.2%) |
| **FNCLCC grade** | |  |
|  | 1 | 18 (4.5%) |
|  | 2 | 116 (28.9%) |
|  | 3 | 268 (66.6%) |

**SUPPLEMENTARY TABLE 2 :** Characteristics of the study population (n=402)

| **Histology** | **N** | **0** | **≥1%** | | **Median IHC Score** |
| --- | --- | --- | --- | --- | --- |
| Soft-Tissue Sarcomas | 402 | 182 | | 220 | 22 CI: 0–285 |
| UPS* | 124 | 59 | | 65 | 2 CI: 0–285 |
| Leiomyosarcoma | 143 | 63 | | 80 | 20 CI: 0–240 |
| Dedifferentiated  Liposarcoma | 21 | 12 | 9 | | 20 CI: 0–190 |
| Myxofibrosarcoma | 74 | 29 | 35 | | 30  CI: 0–180 |
| Rhabdomyosarcoma | 25 | 11 | 14 | | 20 CI: 0–285 |
| Other | 25 | 8 | 17 | | 70 CI: 0–285 |

CI: confidence interval, *UPS: undifferentiated pleomorphic sarcomas,

| **Supplementary Table 3. Antiproliferative activity of 9-ING-41 in soft-tissue sarcoma cell lines** | | | |
| --- | --- | --- | --- |
| **Cell line ID** | **Histological subtype** | **IC_50_ (µM)** | **r^2^** |
| VAESBJ | Epithelioid sarcoma | 0.2271 | 0.975 |
| G402 | Renal leiomyoblastoma | 0.1741 | 0.912 |
| IB112 | Leiomyosarcoma | 0.3210 | 0.987 |
| IB136 | Leiomyosarcoma | 0.3069 | 0.987 |
| IB134 | Leiomyosarcoma | 0.243 | 0.957 |
| IB111 | Dedifferentiated liposarcoma | 0.1390 | 0.956 |
| IB115 | Dedifferentiated liposarcoma | 0.1331 | 0.977 |
| 94T778 | Well-differentiated liposarcoma | 0.2468 | 0.964 |
| 93T449 | Well-differentiated liposarcoma | 0.1721 | 0.977 |
| IB114 | Mixofibrosarcoma | 0.1011 | 0.915 |
| IB116 | Mixofibrosarcoma | 0.5676 | 0.991 |
| IB117 | Mixofibrosarcoma | 0.4819 | 0.907 |
| G401 | Rhabdoid sarcoma | 0.3566 | 0.942 |
| MON | Rhabdoid sarcoma | 0.1058 | 0.856 |
| A204 | Rhabdomyosarcoma | 0.2146 | 0.934 |
| IB128 | Extra-skeletal osteosarcoma | 0.2062 | 0.996 |
| JR588 | Undifferentiated pleomorphic sarcoma | 0.2697 | 0.949 |
| KN473 | Undifferentiated pleomorphic sarcoma | 0.1771 | 0.942 |
| IB106 | Undifferentiated pleomorphic sarcoma | 0.4133 | 0.928 |
| IB152 | Undifferentiated pleomorphic sarcoma | 0.3637 | 0.972 |

**Representative soft-tissue sarcoma cell lines were treated with increasing doses of 9-ING-41 for 72h. After staining of viable cells with Syto24 dye, fluorescence was read with Cytation 3 microplate reader and IC50 determined with Graphpad software (n=3)**

**SUPPLEMENTARY FIGURES**


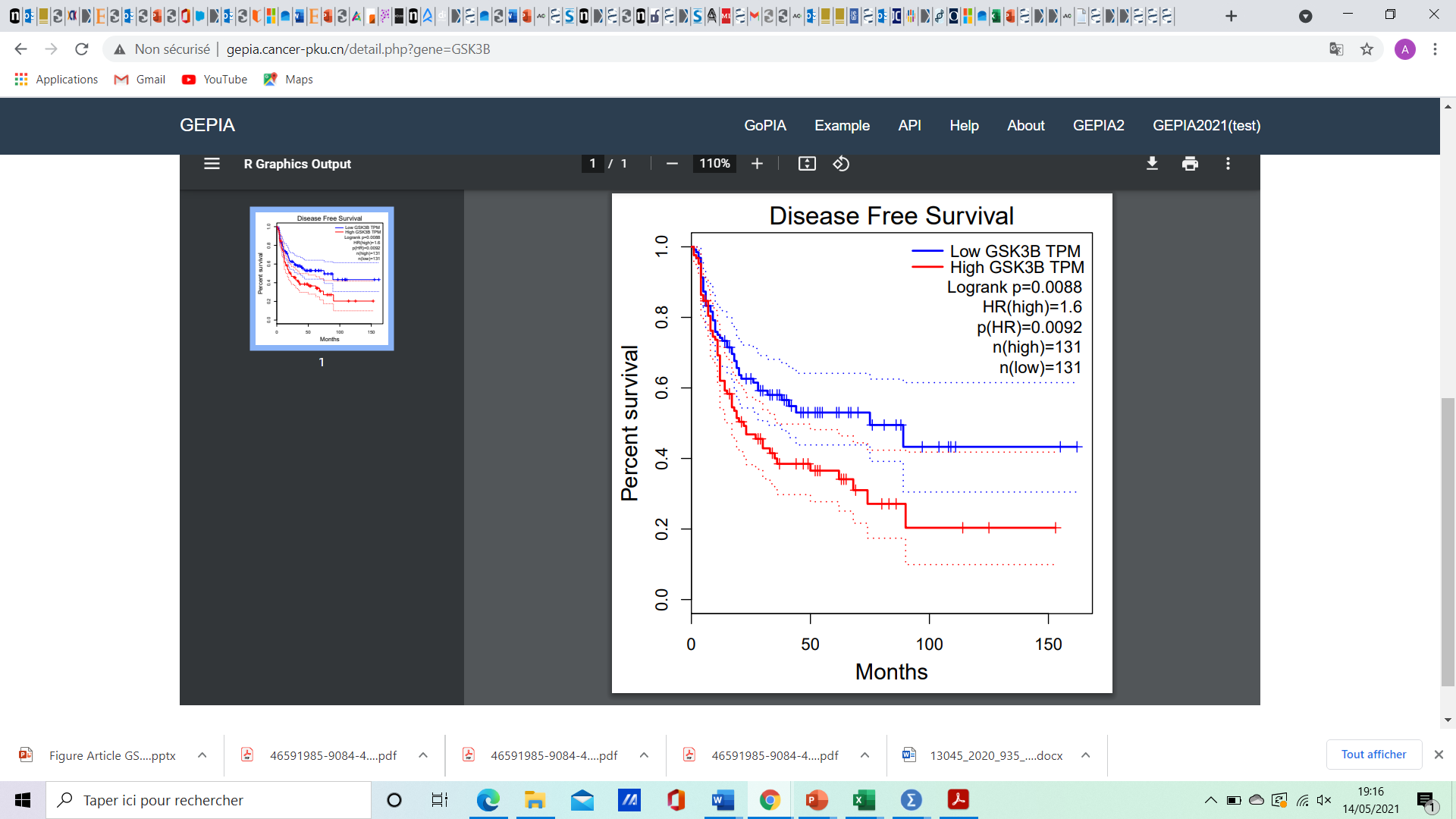


**Supplementary Figure 1.** Kaplan-Meier curves of disease-free survival according to *GSK3β* gene expression in 262 soft-tissue sarcomas (<http://gepia.cancer-pku.cn/index.html>). Dotted line corresponds to the 95% confidence interval.


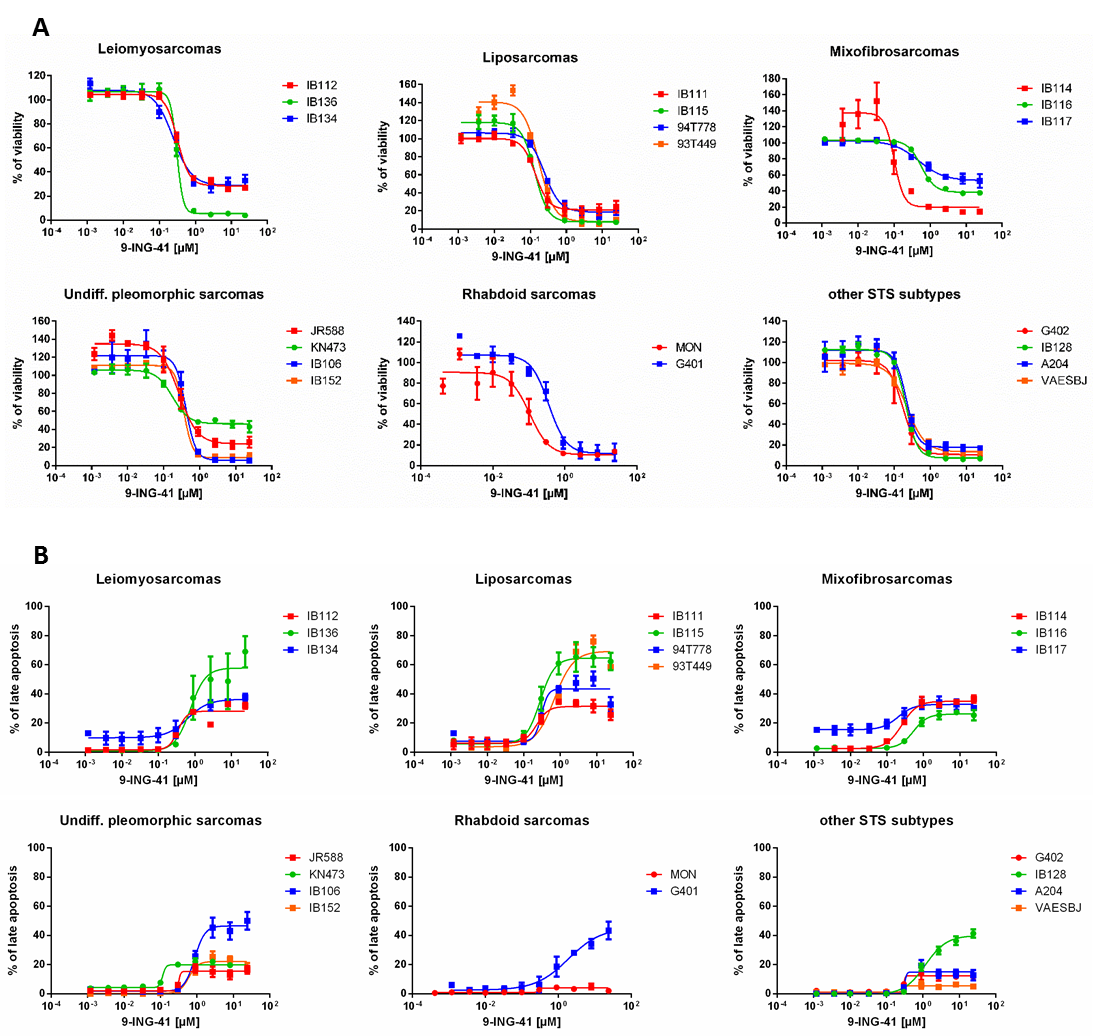


**Supplemental Figure 2. Cell viability and apoptosis curve after 9-ING-41 treatment in soft-tissue sarcoma cell lines.** Soft-tissue sarcoma cell lines were treated with increasing doses of 9-ING-41 for 72h. After staining of viable cells with Syto24 dye (A) or late apoptotic cells with propidium iodide (B), fluorescence were read with Cytation 3 microplate reader and IC50 determined with Graphpad software based on the viability curve (n=3) (See IC50 values in supplementary Table 3).


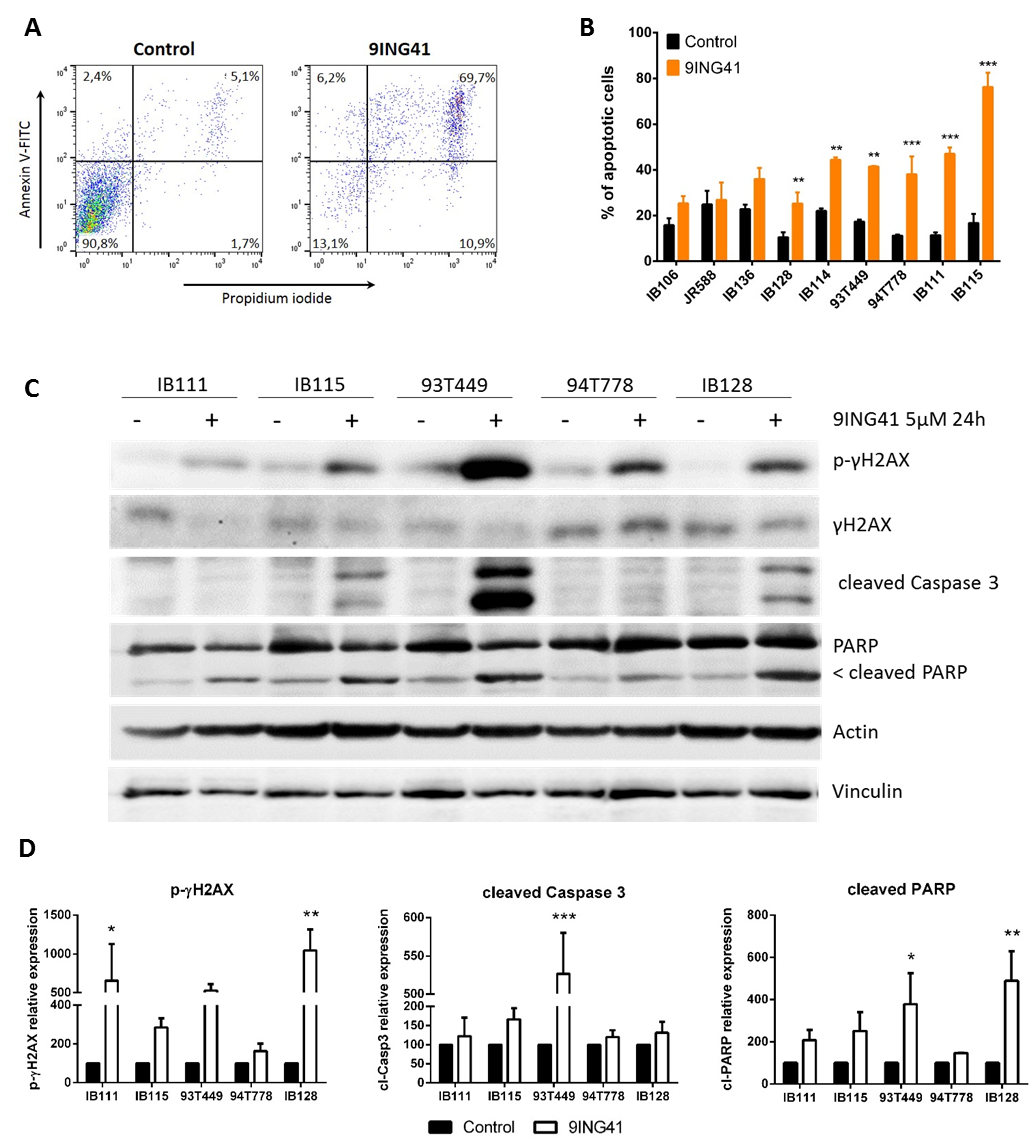


Supplementary Figure 3. Cytotoxic effect of GSK3β inhibitor 9-ING-41 in soft-tissue sarcoma cells. A: Annexin-V FITC vs propidium iodide (PI) plots from the IB115 cells shows the populations corresponding to viable (Annexin V–PI–), early (Annexin V+ PI–), and late (Annexin V+ PI+) apoptotic cells after 48 h of treatment with 9-ING-41 at 0.5µM. B: Quantification of apoptotic cells in STS cell lines after 48 h of treatment with 9-ING-41 at 0.5µM (n=3; **p<0.01 ***p<0.001; Multiple t tests). C: Western blot of apoptotic marker after 24h of 9-ING-41 5µM treatment. D: Quantification of previous western blot using ImageJ software (n=2). Phospho-γH2AX, cleaved Caspase3 and PARP are upregulated accordingly with the apoptosis results.

**
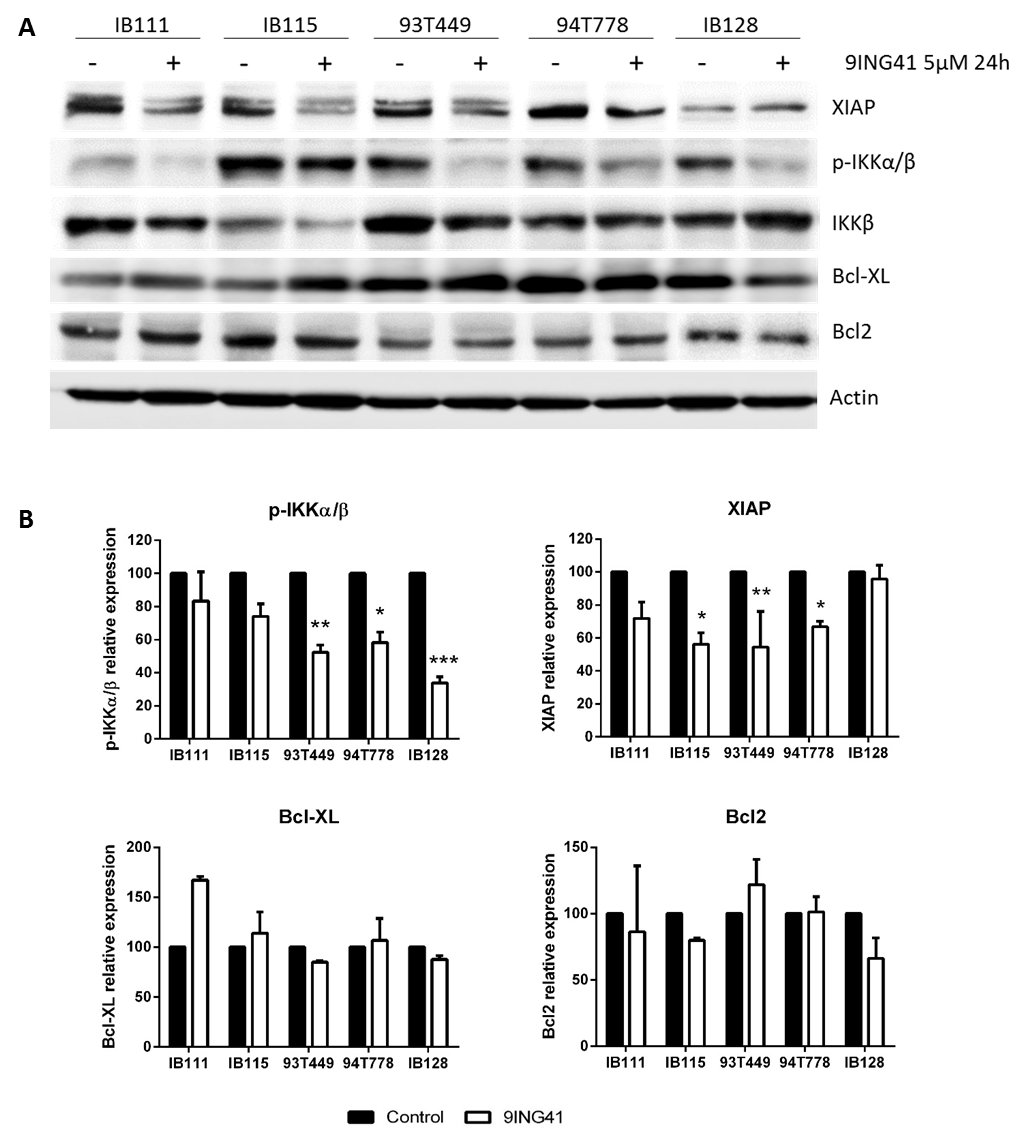
**

**Supplementary Figure 4. NFκB pathway regulated by GSK3β inhibitor 9-ING-41 in soft-tissue sarcoma cells. A:** Western blot of a panel of protein regulated by GSK3β. After 24h of 9-ING-41 5µM treatment, XIAP and phospho-IKKα/β are downregulated while Bcl2 and Bcl/XL are not. B: Quantification of previous western blot using ImageJ software (n=2, **p<0.01 ***p<0.001; Multiple t tests).
